# Electroanalytical Determination of Cysteine Using the Electrodes Based on Ternary Silver-Copper Sulfides

**DOI:** 10.3390/s18113753

**Published:** 2018-11-02

**Authors:** Nives Vladislavić, Ivana Škugor Rončević, Maša Buljac, Slobodan Brinić, Denis Krivić, Marijo Buzuk

**Affiliations:** 1 Department of General and Inorganic Chemistry, Faculty of Chemistry and Technology, University of Split, 21000 Split, Croatia; nives@ktf-split.hr (N.V.); skugor@ktf-split.hr (I.Š.R.); brinic@ktf-split.hr (S.B.); 2 Department of Environmental Chemistry, Faculty of Chemistry and Technology, University of Split, 21000 Split, Croatia; masa@ktf-split.hr; 3 Faculty of Chemistry and Technology, University of Split, 21000 Split, Croatia; dk9562@ktf-split.hr

**Keywords:** cysteine, determination, silver-copper sulfides, amperometry, electrochemical, impedance spectroscopy, FTIR, electroanalysis

## Abstract

The amperometric determination of cysteine, using an electrode based on ternary silver-copper sulfide, is presented. Electrochemical characterizations (using cyclic voltammetry) of three electrode materials revealed that the electrode based on the material that consists of jalpaite (Ag_3_CuS_2_), doped with a small amount of metallic silver, has the best electrocatalytical performance for cysteine oxidation. For the amperometric determination, 0.142 V at pH 5 and 0.04 V at pH 7 vs. Ag/AgCl, related to the electrocatalytical oxidation of thiol group, were chosen. Electrochemical impedance spectroscopy together with Fourier transform infrared spectroscopy (FTIR) revealed that oxidation takes place on the electrode surface with fouling effect, which does not affect a wide linear working range between 1 μM and 100 μM. Sensitivities, at pH 5 and pH 7, are calculated to be 0.11 μA μM^−1^ and 0.10 μA μM^−1^, respectively. The detection limits were determined to be 0.036 μM and 0.024 μM for pH 5 and pH 7, respectively. In the presence of uric acid, folic acid, ascorbic acid, and glucose no interference was noticed. This electrode showed remarkable stability and excellent reproducibility. The electrode was exploited for the determination of cysteine in a dietary supplement with the excellent recoveries.

## 1. Introduction

Cysteine has an important role in biochemical processes, as well as in environmental systems [1,2]. Cysteine, as non-essential amino acid, can be found in protein food, various cosmetics, and pharmaceutical products [3]. Determination and quantification of cysteine or its derivates in biological fluids (urine, blood, etc.) is very important, since cysteine plays a role in several diseases such as depigmentation of hair, growth disorder in children, liver damage, as well as in some pathological conditions such as Alzheimer’s or Parkinson’s disease [4,5,6]. It was shown that cysteine is a major extracellular antioxidant and that it plays an important role in various physiological antioxidant systems. In addition, cysteine is involved in the formation of reactive sulfur species (RSS) and in appearance of a specific type of oxidative stress, which is associated with the oxidation of the pair cysteine/cystine, called disulfide stress [7].

Thus, up to now the determination of cysteine has attracted considerable attention. Although many techniques have been developed for the determination of cysteine, such as high-performance liquid chromatography (HPLC) [8,9,10,11], spectrophotometry [12,13], or gas chromatography/mass spectrometry [14], the most exploited and reported techniques are electrochemical due to a favorable redox activity of its functional groups. In comparison with the techniques mentioned above, electrochemical sensors can be constructed in a form of point-of-care systems since they are cheap, fast, and easy to use.

Mainly, electrochemical study of cysteine is based on its behavior, under anodic conditions (e.g., oxidation of thiol group), at various metal electrodes (gold, mercury, platinum, graphite, etc.). Also, articles referring to this topic were quoted by Hager and Brolo [15]. However, the electrochemical behavior of cysteine on the above-mentioned materials is quite meager [16,17], or it is characterized with a high positive overpotential [18]. The main method for overcoming these issues is to use new materials [16] via modification of various carbon-based materials. In addition, detailed information concerning recent achievements in electrochemical study of cysteine, using modified electrodes, was summarized in several review articles [19,20,21,22]. However, some electrode modifications require the confining of appropriate mediators into matrix, which can make the construction of an electrode complicated. In addition, often the sensors based on redox mediators tend to experience difficulty in differentiating similarly constructed thiols [17], which can hamper the possibility of constructing the commercially sensor system.

To overcome above-mentioned problems, a sensor based on a crystal material seems to be promising. The idea came from the known fact that some crystal materials (mostly based on silver salts) were used in potentiometric determination of thiols [23,24,25,26]. Also, the commercial ion-selective electrode for Cu^2+^ ions (based on silver-copper sulfide mineral—jalpaite) was used in the study of the mechanism and rate of the “self-oxidation” of cysteine in the presence of Cu^2+^ [27]. For this purpose, the well-known catalytic role of copper cations, in the oxidation of cysteine, was used [28,29,30]. However, the latter hampers potentiometric determination of cysteine by materials based on jalpaite (or other copper-consisting salts). In addition, use of the dynamic electrochemical techniques is limited by insufficient electrical conductivity of the jalpaite. Recent reports, concerning such various ternary silver-copper sulfides with sufficient electrical conductivity [31], show that it seems to be promising for use in the electrochemical determination of cysteine using dynamic techniques.

The problem of insufficient electrical conductivity of crystal materials (for example CuS) in development of various electrochemical sensors have been aim of many studies. Among the reported approaches are several papers that deserve attention: electrochemical immunosensor based on electrooxidation of catechol on CuS/reduced graphene oxide nanocomposite [32], sensors for detection of H_2_O_2_ by CuS/reduced graphene oxide composites [33], electrochemical sensors for determination of caffeine by copper sulfide carbon paste electrode [34], electrochemical immunoassay of myoglobin based on copper sulfide-functionalized molybdenum disulfide [35] or amperometric glucose sensor [36], electrochemical sensors for detection of glucose by CuS microflower [37], electrochemical sensing of picric acid with a copper sulfide-modified glassy carbon electrode [38], electrochemical determination of nitrite by CuS-MWCNT nanocomposite-based sensor [39], and amperometric sensors for determination of glucose and H_2_O_2_ by cuprous sulfide nanorods [40]. Also, silver-based salts (as Ag/Ag_2_S modified carbon nanotube) were introduced as favorable for the simultaneous detection of dopamine and ascorbic acid [41,42] or for the detection of hemoglobin [43].

Two reports on the electrochemical behavior of metal-based sulfides in the presence of cysteine were published by Pei et al. [44,45]. In these studies, authors modified the surface of glassy carbon electrode with different sulfides (Ag_2_S, Ag_2_S/Cu_2_S, CuS and Cu_1.1_Fe_1.1_S_2_). The study was performed in the presence of cysteine and within two solutions: NaCl solution and PBS buffered solution. In the presence of cysteine, CuS modified glassy carbon electrode exhibited pure electroactivity. When glassy carbon electrode was modified with Cu_1.1_Fe_1.1_S_2_, irreversible anodic processes were observed in both solutions. Modification of glassy carbon electrode with Ag_2_S or Ag_2_S/Cu_2_S resulted in semi-reversible behavior of both modified electrodes, which showed that prepared electrodes have a potential towards cysteine determination. Another study, which is concerned with the electrochemical behavior of cysteine using metal sulfide-based electrode, was reported by Wang et al. [46].

In this study, we focused on the investigation of the electrochemical behavior of cysteine on three different crystal electrodes. This was done to obtain the information about the possibility for their use in the electrochemical determination of cysteine. The electrodes were prepared using three different materials (based on silver-copper sulfides). The prepared materials were characterized by X-ray diffraction (XRD) analysis [31]. Electrochemical impedance spectroscopy (EIS) together with FTIR was used to get an insight into the processes that take place on or near the electrode surface. The most promising electrochemical behavior, towards cysteine, was obtained with the electrode material consisting of jalpaite and metallic silver. The electrode based on this material was used for the amperometric determination of cysteine. The analytical response of this electrode was tested at various pH values and in a solution that contained various interferences. In addition, the electrode was exploited for the determination of cysteine in a dietary supplement with excellent recovery values.

## 2. Materials and Methods

### 2.1. Chemicals and Solutions

All chemicals were of analytical reagent grade. All solutions were prepared with redistilled water. Acetate buffer (pH 5; 0.1 M) was prepared by mixing of previously prepared solutions (0.1 M) of potassium acetate and acetic acid (0.1 M), all purchased from Kemika (Zagreb, Croatia). Potassium dihydrogen phosphate and potassium hydrogen phosphate (all purchased from Sigma-Aldrich, St. Louis, MO, USA) were used for preparation of phosphate buffers (0.1 M; pH 7). Stock solution of the copper nitrate (1 × 10^−3^ M) was prepared by dissolution of Cu(NO_3_)_2_ × 5H_2_O (Sigma-Aldrich, Inc.) in potassium nitrate (Kemika, Zagreb, Croatia) solution (0.1 M). The cysteine solution (Merck, Kenilworth, NJ, USA) and the food supplement solution (NAC-Twinlab^®^ dietary supplement, Hauppauge, NY, USA) were prepared daily by its dissolution in redistilled water previously deaerated (with N_2_). Uric acid, ascorbic acid and glutathione were purchased from Sigma-Aldrich (St. Louis, MO, USA), while glucose was obtained from Kemika, Zagreb, Croatia.

### 2.2. Electrode Fabrication

Preparations of three electrode materials were done according proposed method [31]. Electrode materials were obtained by co-precipitation of Ag^+^ and Cu^2+^ with sulfide at various mole ratio as it follows: Material “AC0.5S” at mole ratio Ag^+^ and Cu^2+^:S^2−^ = 1:0.5, Material “ACS” with mole ratio of Ag^+^ and Cu^2+^:S^2−^ = 1:1 and material “AC1.8S” with mole ratio of Ag^+^ and Cu^2+^:S^2−^ = 1:1. Electrodes (as pellets) were prepared by pressing of the powder electrode material at 5000 kg cm^−3^: Obtained electrode thickness was 1 mm and the surface area was 1.3 cm^2^. Electric contact was achieved by fixing pellets onto stainless steel sticks using conductive silver epoxy resin (SPI Supplies Division, West Chester, PA, USA). Finally, fixed pellets were sealed by epoxy resin. After drying, electrodes were abraded with fine emery paper and polished with alumina powder down to 0.05 μm. The electrodes are named after the materials they are made of.

### 2.3. Apparatus and Measurements

Electrochemical measurements (cyclic voltammetry and amperometry) were carried out with potentiostat (Autolab PGSTAT 302N, Metrohm, Autolab B.V., Utrecht, The Netherlands), connected to PC and driven by GPES 4.9 Software (Eco Chemie, Metrohm, Autolab B.V., Utrecht, The Netherlands). An electrochemical cell with three electrodes was used with Radiometer XR300 Ag/AgCl 3M electrode as reference electrode, Pt plate as auxiliary and pressed electrodes as working electrode. All experiments were carried out at 25 °C. Three cycles were recorded and in results third cycles are presented. Cyclic voltammograms were recorded in quiescence solution started towards cathodic potential at the scan rate of 25 mV s^−1^, while amperometric measurements were performed under stirring condition. All electrochemical measurements were performed in solutions containing 90 mL 0.1 M KNO_3_ + 10 mL of appropriate buffer.

The EIS measurements were performed at the open circuit potential (OCP) with an ac voltage amplitude of ±10 mV in the frequency range from 100 kHz to 0.05 Hz. Measurements were carried out using a Solartron SI 1287 electrochemical interface and a Solartron SI 1255 frequency response analyzer. The EIS data were fitted using the complex non-linear least squares fit analysis by Zview software (Ametek, Berwyn, PA, USA). The numerical values of impedance parameters were determined with a standard deviation χ^2^ of the order of 10^−5^, and the relative error of each element was less than 5%. An electrochemical cell with three electrodes was used with Radiometer XR300 Ag/AgCl 3 M electrode as reference electrode, Pt plate as auxiliary and pressed electrodes as working electrodes.

The FTIR spectra were recorded in the 4000–650 cm^−1^ region using Horizontal Attenuated Total Reflectance (HATR) method on a Perkin-Elmer Spectrum One FTIR spectrometer. FTIR spectra of electrodes were recorded after recording of CVs at 1 × 10^−4^ M cysteine at pH 7.

## 3. Results and Discussion

Information about phase composition of the prepared materials was previously reported [31]. XRD pattern for the material named “AC0.5S” indicate presence of metallic silver, Ag_1.2_Cu_0.8_S and CuAgS (Stromeyerite). Material “ACS” mainly consisted of Ag_3_CuS_2_ (jalpaite) and metallic silver, while crystal phases of Ag_1.2_Cu_0.8_S and Ag_3_CuS_2_ (jalpaite) was found in material “AC1.8S”.

To explore electrochemical behaviors of prepared electrodes in the presence of cysteine at three pH values (5, 7 and 9), cyclic voltammetry was used. It must be emphasized that for all three electrodes, results obtained at pH 9 were not reliable and reproducible due to improved dissolution of electrode materials at higher pH values, thus results are not shown.

### 3.1. Voltammetric Characterization

**Figure 1:**
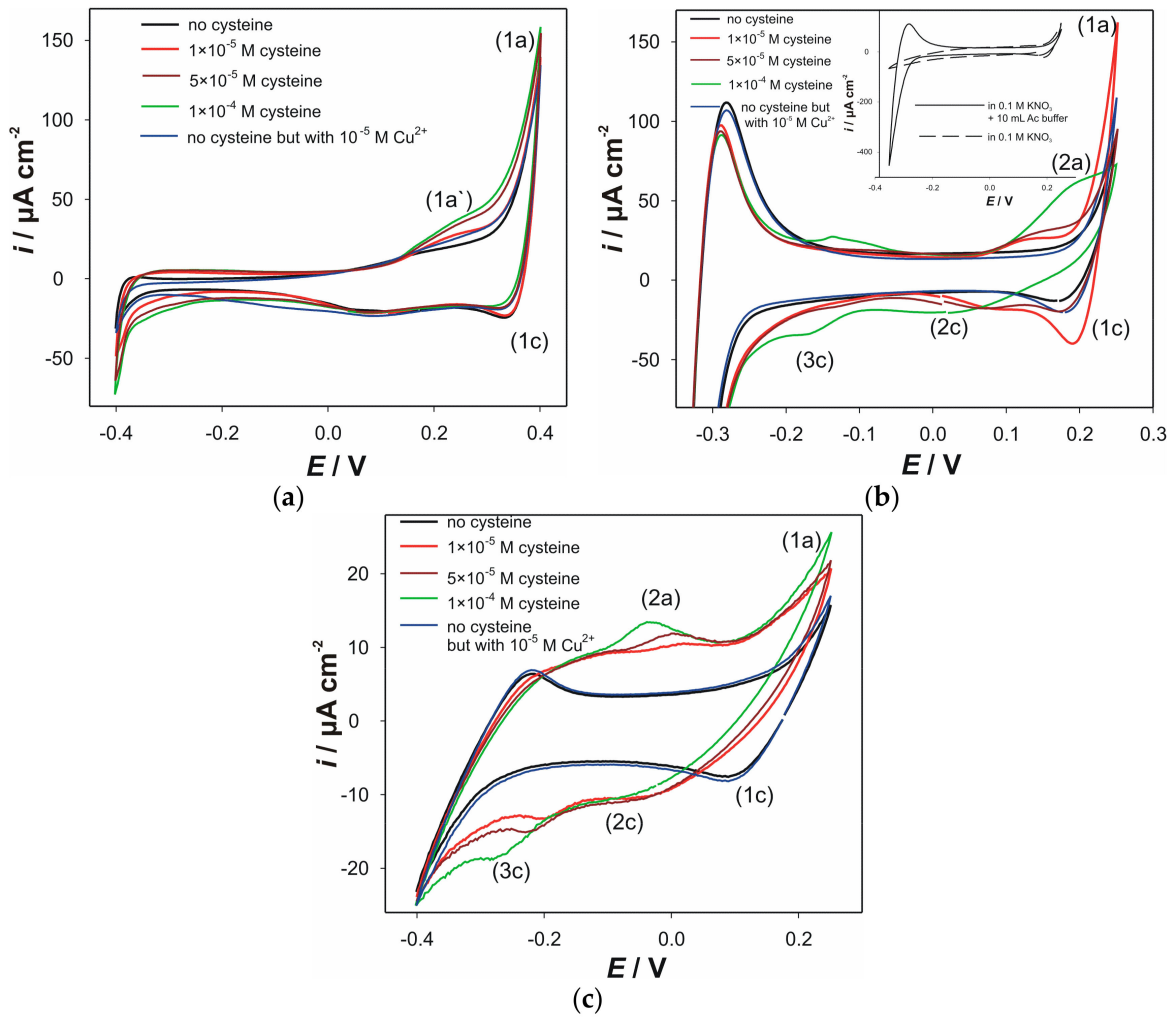
CVs obtained in 90 mL 0.1 KNO_3_ + 10 mL 0.1 M acetate buffer (pH 5) without and with the presence of various cysteine concentrations (successive cysteine addition): (**a**) electrode “AC0.5S”; (**b**) electrode “ACS”; (**c**) electrode “AC1.8S”. Inset in Figure 1b: Influence of the acetate buffer addition on the CV of the electrode “ACS”. Scan rate 25 mV s^−1^.

Cyclic voltammograms (CVs) obtained with the electrodes “AC0.5S”, “ACS” and “AC1.8S”, at pH 5, in the presence of various cysteine concentrations, are shown in Figure 1. CVs for all three electrodes, recorded in the pure electrolyte solution, revealed the presence of a large oxidation current peak (1a) and a corresponding cathodic current peak (1c). The current peak (1a) corresponds to anodic oxidation of the electrode material according the reaction (1) [47]. Furthermore, formed cuprous ions can be oxidized into cupric ions, which can influence the current peak (1a). The latter will have an impact on the current peak (1c). The cathodic current peak (1c) can be attributed to the reduction of the electrode material according to the reverse reaction (1) or, more possibly, to the cathodic reduction of copper ions. Noteworthy, in the case of the electrode “ACS”, large current peaks (around −0.3 V) were obtained in acetate-buffered solution (Inset of Figure 1b). These current peaks correspond to the redox activity of the buffer acetate group. This phenomenon did not affect the electrochemical behavior of cysteine. As presented in Figure 1c, the electrode “AC1.8S” has lower redox activity, due to the pure conductivity of this material, which is a consequence that rises from the lack of metallic silver in the material “AC1.8S”.

Cu^2^+ + Cu_2_S *+ 4H_2_O ⇆ CuSO_4_ + 2Cu^+^ + 8e^–^ + 8H^+^ (1)

* as AgCuS in the material “AC0.5S” or as Ag3CuS in the materials “ACS” and “AC1.8S”.

Upon the addition of cupric ions (blue line), several changes in the electrochemical behaviors of the electrodes “AC0.5S” and “ACS” can be observed. On the contrary, the CV of the electrode “AC1.8S” did not shown any changes upon the addition of cupric ions. In the presence of the cupric ions the increase in the anodic current peak (1a′) observed at the electrode “AC0.5S” (Figure 1a) indicates feasible oxidation of the electrode material, according to the reaction (1). This behavior was not observed at the electrode “ACS”. However, at the CV obtained from the electrode “ACS”, cathodic current peak (1c) becomes more prominent in the presence of cupric ions, which can be attributed to the cathodic reduction.

The addition of cysteine was carried out in the solution containing 1 × 10^−5^ M Cu^2+^ at pH 5. It resulted in several changes in the recorded CVs (Figure 1). CVs obtained at the electrode “AC0.5S” (Figure 1a) shows that the anodic current peak (1a′) increases with the addition of cysteine. This clearly indicates that presence of cysteine facilitates the oxidation of the electrode material due to complexation of cuprous ions (according to reactions (2) and (3)), thus producing a shift in the equilibrium towards products (1).

RS^−^ + Cu^+^ ⇆ RSCu (2)

RSCu + Cu^+^ ⇆ (RS^−^)Cu^+^(RS^−^) (3)

Secondly, increasing the concentration of cysteine slightly decreases the current peak (1c), since the copper ions are present in the complex form.

Upon the addition of cysteine, more evident changes can be observed at the electrode “ACS”. As it is shown (Figure 1b), the addition of cysteine (1 × 10^−5^ M) resulted in the occurrence of the oxidation signal at +0.1 V (current peak (2a)). Also, significantly enhanced dissolution of the electrode material can be noticed (red line-increasing of the anodic current peak (1a)). This can be attributed to the instant complexation of the added cysteine with the copper ions that originates from the anodic reaction. Interestingly, the latter was not followed by decrease in the cathodic current peak (1c). This suggests formation of the non-stabile complexes (at the 1 × 10^−5^ M of cysteine). Accordingly, an increase in the cathodic current peak can be observed. By further addition of cysteine (5 × 10^−5^ M; brown line) the cathodic current peak (1c) becomes less prominent, as the copper cations are now tightly bound (to cysteine) in the complex, according to reactions (2) and (3). Subsequently, the additional cathodic current peak (2c) occurs. This current peak corresponds to cathodic reduction of the copper ions from its complex form. This current peak becomes wider with the addition of cysteine. Increasing the concentration of cysteine decreases both anodic and cathodic current peaks ((1a) and (1c)), thus indicating that corresponding reaction becomes hampered. This is preceded by an increase of the anodic current peak (2a) with the addition of cysteine. This is related to the formation of cystine on the electrode surface (reactions (4) and (5)) that passivizes it. Also, at the concentration of 1 × 10^−4^ M of cysteine (green line), increase of the cathodic current (3c) can be attributed to the processes that take place in bulk of the formed layer. This being mostly related to the reduction of various complex forms (copper cysteinate), or even electrochemical reduction of formed cystine.

RSCu ⇆ Cu^2+^ + e^–^ + RS^−^ (4)

2RS^−^ ⇆ RSSR + 2e^−^ (5)

or depending on pH: 2RSH ⇆ RSSR + 2e^−^ + 2H^+^

CVs of the electrode “AC1.8S”, obtained in the presence of the cysteine (Figure 1c) suggest, to some extent, pseudocapacitive behavior. This behavior can be related to the electrochemical processes that take place in the formed surface layer that mostly consists of the produced cystine and cuprous cysteinate complex. We propose tentative mechanism for the formation of this surface layer, as well as the layer’s role in the observed pseudocapacitive behavior. This layer was formed during three CV cycles, as it follows: during the first cycle (towards cathodic potentials) electrochemical reduction of cupric into cuprous cations. Simultaneously, the chemical reduction of cupric ions according to reaction (6) occurs near the electrode surface.

RS^−^ + Cu^2+^ ⇆ Cu^+^ + RS (6)

Produced thiol radical (RS) will recombine to cystine according to reaction (7)

RS + RS ⇆ RSSR(7)

Also, added cysteine reacts with formed cuprous cations (reaction (6)) to form cysteinate complex according to reaction (2). The products of the reactions (2) and (7) form a layer on the surface of the electrode “AC1.8S”. Pseudocapacitive behavior can be attributed to the processes that take place within the formed surface layer. As shown in Figure 1c, the anodic current peak (2a) becomes more prominent with an increase of the cysteine concentration. This behavior arises from the electrochemical oxidation of cystine, as presented by the reaction (5). Furthermore, this reaction is preceded by the anodic oxidation of cuprous cysteinate (reaction (4)), as it affects the pseudocapacity. The product of the reaction (4) is cupric ion. Finally, it gets involved in the reaction (6) to produce cuprous cysteinate according to reaction (2). Simultaneously, during cathodic cycle, production of cuprous cysteinate is also facilitated by the electrochemical reduction of cupric ions. All these processes contribute to pseudocapacitive behavior. In addition, the current peak (2c) can be related to the same process described for the electrode “ACS”. Also, the increase of the cathodic current (current peak (3c)) suggests that electrochemical reduction of the formed cystine takes place.

**Figure 2:**
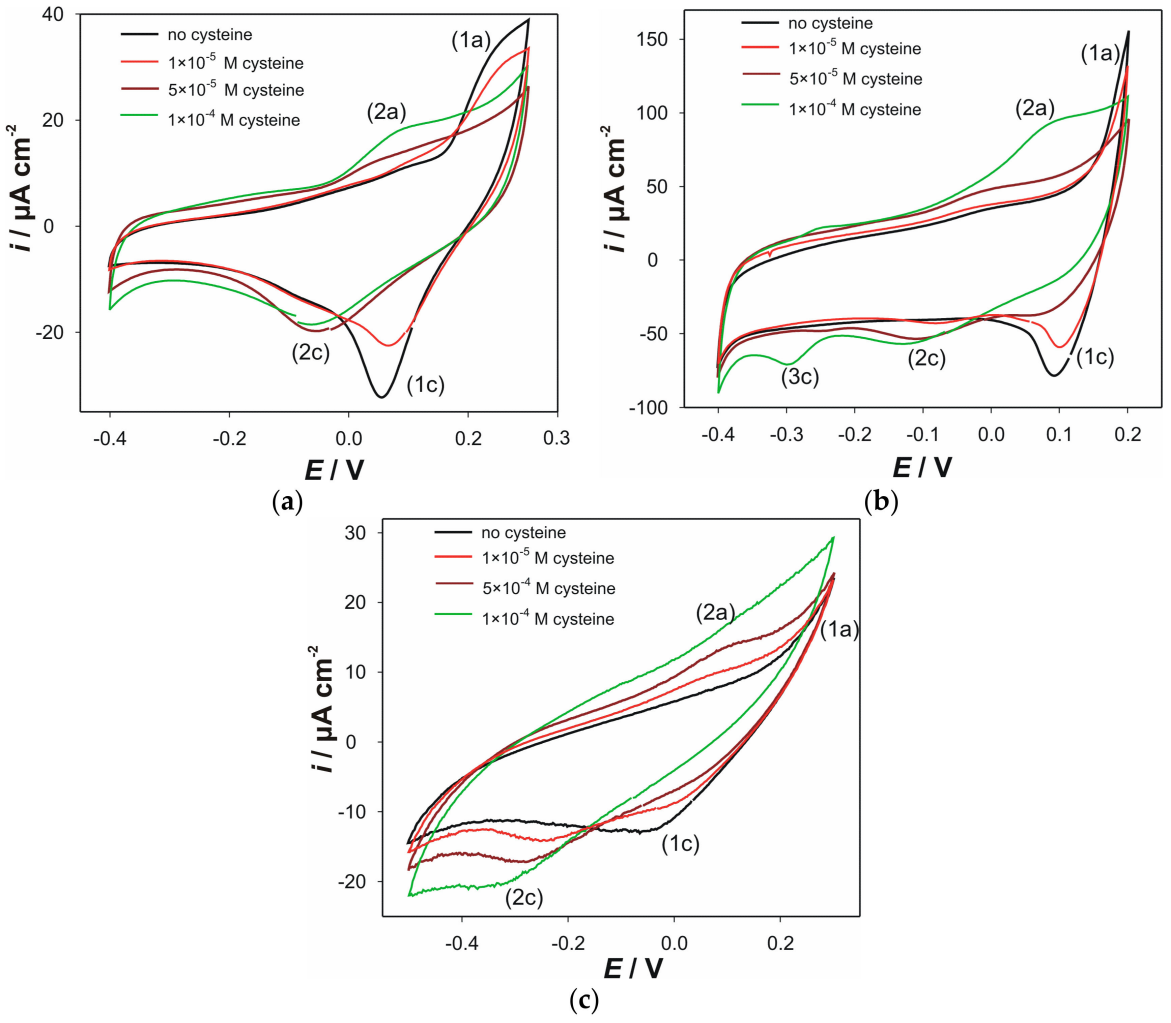
CVs obtained in 90 mL 0.1 KNO_3_ + 10 mL 0.1 M phosphate buffer (pH 7) without and in the presence of various cysteine concentrations (successive addition of cysteine): (**a**) electrode “AC0.5S”; (**b**) electrode “ACS”; (**c**) electrode “AC1.8S”_._ Scan rate 25 mV s^−1^.

CVs of electrodes “AC0.5S”, “ACS” and “AC1.8S” at pH 7, in the presence of various cysteine concentrations, are shown in Figure 2. Measurements in the presence of cupric ions were not carried out due to complexation capacity of media towards metal cations.

At pH 7 (Figure 2a), anodic dissolution (current peak (1a)) of the electrode “AC0.5S” can be observed. Consequently, corresponding cathodic current peak (1c) was obtained. This current peak corresponds to the reduction of the copper ions from its hydroxyl complexes.

Upon the addition of cysteine addition, in cyclic voltammograms of the electrode “AC0.5S” at pH 7, can be observed (Figure 2a). Firstly, the disappearance of the anodic current peak (1a) with the addition of cysteine (green line) can be observed. This is followed by appearance of a new anodic current peak (2a). Similar to the measurement performed at pH 5, this current peak can be attributed to the oxidation of the thiol group of cysteine (reaction (5)). Produced cystine causes fouling effect at the electrode surface. Consequently, accumulated cystine can hamper an anodic dissolution of the electrode material. This is in accordance with the decrease in the anodic current peak (1a). This behavior (but with semi-irreversible character), on Ag2S/Cu2S crystal modified GCE, was also reported by Pei et al. [44]. Consequently, electrochemical reduction of the copper species becomes reduced owing to the formation of copper cysteinate complexes. Thus, decrease and shift in the cathodic current peak (1c) towards (2c) can be observed.

The influence of various cysteine concentrations on cyclic voltammograms of the electrode “ACS” are shown in Figure 2b. Similar to the behavior observed at pH 5, the current peaks (1a) and (1c) correspond to the oxidation of the electrode material. This is followed by the reduction of the oxidation products. The addition of cysteine (5 × 10^−4^ M) produced the anodic signal that started to occur around −0.05 V (current peak (2a)). This signal becomes more prominent at the higher cysteine concentration (1 × 10^−4^ M). As it was observed at pH 5, this oxidation current peak corresponds to the anodic oxidation of copper cysteinate, as well as to the anodic oxidation of cysteine. The addition of cysteine also decreases and shifts the cathodic current peak (1c) towards more cathodic potentials (current peak (2c)). This can be attributed to the reduction of copper ions from its complexes with cysteine. Redox activity, observed at cathodic branch of voltammogram (current peak (3c)), is probably related to reduction of cystine.

Cyclic voltammograms obtained at pH 7, at various cysteine concentrations, for electrode “AC1.8S” are shown in Figure 2c. Due to additional competitive reactions towards copper species in the basic media, the electrochemical processes are scarcely distinguishable. However, some similar processes, such as the formation of cuprous cysteinate, preceded by the electrochemical reduction of cupric into cuprous cations, and the reduction of cystine (beyond −0.2 V) can be “recognized”. Also, the oxidation processes that take place between 0.0 and +0.2 V correspond to the proposed reactions (4) and (5).

### 3.2. Electrochemical Impedance Spectroscopy of the Electrodes

As obtained CVs for the electrodes “ACS” and “AC1.8S” indicate formation of the surface layer, additional experiments, concerning surface processes, were carried out. To obtain the information about the processes at the surface of the electrodes, such as fouling effect, the EIS was used. Electrochemical impedance spectra of the electrodes were recorded in the presence of 1 × 10^−5^ M Cu^2+^, at the various cysteine concentrations.

**Figure 3:**
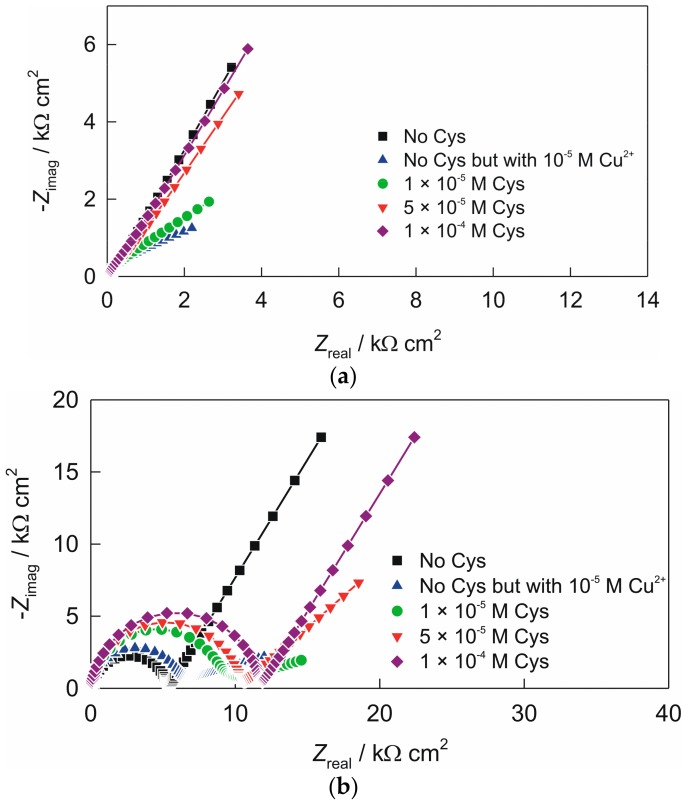
Nyquist plots recorded in 90 mL 0.1 KNO_3_ + 10 mL 0.1 M acetate buffer (pH 5), without and with the presence of the various cysteine concentration (by successive addition of cysteine in the solutions that were spiked with 1 × 10^−5^ M Cu^2+^): (**a**) electrode “ACS”; (**b**) electrode “AC1.8S”. Frequency range: 100 kHz–0.05 Hz.

Recorded Nyquist plots at pH 5, for the electrode “ACS” and “AC1.8S” are shown in Figure 3.

**Scheme 1:**
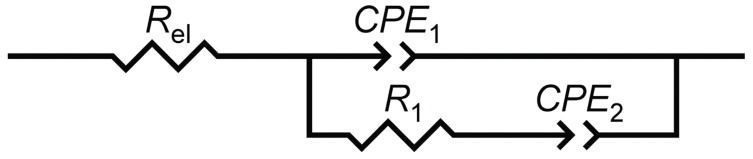
Equivalent electrical circuit model used for analysis of impedance spectra of electrodes “ACS” and “AC1.8S”.

In Nyquist plot, two more or less separated time constants can be observed for both tested electrodes: time constant or capacitive loop in the high frequencies range and time constant that point towards the presence of a diffusion process. EIS data recorded were modeled by the electrical equivalent circuit (EEC) with two time constants shown in Scheme 1. In the EEC, *R*_el_ is the electrolyte resistance, constant phase element, *CPE*_1_ attributed to the double layer capacitance, *R*_1_ to the charge-transfer resistance and *CPE*_2_ represent a dimensional diffusion through a layer of finite thickness or the (penetrability) penetration depth of the ac signal across pores of finite length. The numerical values of impedance parameters obtained by fitting procedure are listed in Table 1. The constant phase element (*CPE*) was used instead of non-ideal capacitance [48]. Its impedance is equal to *Z*(*CPE*) = [*Q*(*jω*)*^n^*]^−1^, where *Q* is the frequency independent parameter, ω is the angular frequency, and n is the CPE power. When *n* = 1, *Q* represents the pure capacitance, while for *n* ≠ 1 the system shows behavior that has been attributed to the surface heterogeneity [49], or to the continuously distributed time constants for charge-transfer reactions [50]. For a specific value of *n*, *Z*(*CPE*) describes the resistance (*n* = 0), capacity (*n* = 1), inductance (*n* = −1) and the diffusion processes (*n* = 0.5).

Recorded Nyquist plots for the electrode “ACS”, at pH 5, are presented in Figure 3a. By addition of cupric cations charge-transfer resistance (R_1_) becomes less prominent. However, successive addition of cysteine results in the increase of (R_1_) and in diffusion-controlled processes.

Recorded Nyquist plots of the electrode “AC1.8S” at pH 5, are shown in Figure 3b. As compared to the spectrum recorded without the presence of cysteine, Nyquist plots of the electrode “AC1.8S”, when cysteine was added exhibit higher capacitive semi-circle as well as greater overall impedance. It is also evident that the capacitive semi-circle increases with an increase in the cysteine contractions. As it shown in Figure 3b and Table 1, significant increase of the charge-transfer resistance, (R_1_) at the electrode/solution interface, with increase in the cysteine concentration was observed. This is due to enhanced formation of cystine and cysteinate. Increase in cysteine concentrations decrease the value of the electrochemical double layer capacitance. This indicates an increment in thickness of the surface film as well as the structural changes within the film. The diffusion element (Q_2_) decreases in the same way.

**Figure 4:**
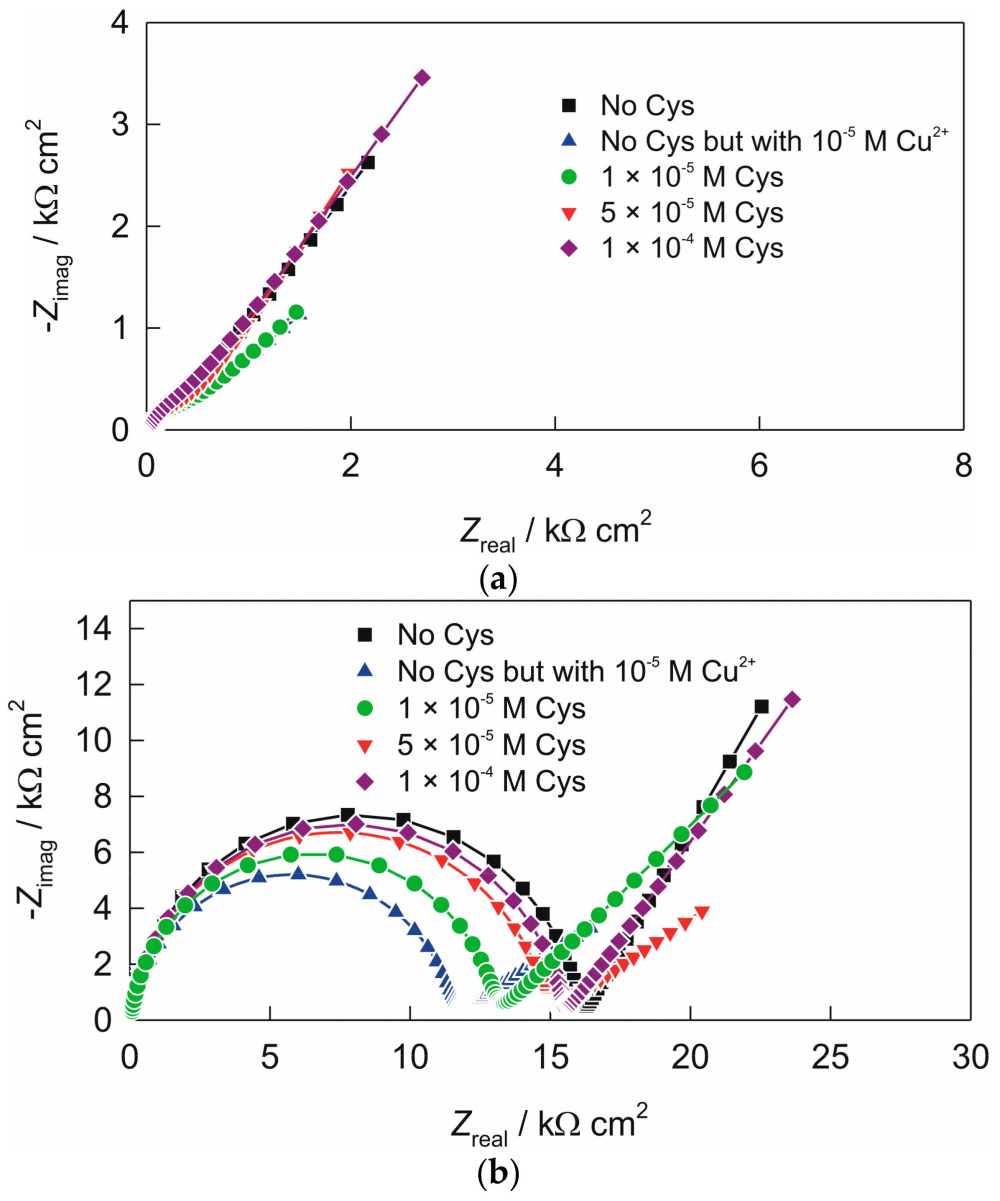
Nyquist plots recorded in 90 mL 0.1 KNO_3_ + 10 mL 0.1 M phosphate buffer (pH 7), without and in the presence of various cysteine concentrations (by successive addition of cysteine in the solutions that were spiked with 1 × 10^−5^ M Cu^2+^): (**a**) electrode “ACS”; (**b**) electrode “AC1.8S”. Frequency range: 100 kHz–0.05 Hz.

As in the case when pH was 5, impedance spectra were recorded at pH 7, to obtain additional information about the surface processes. Obtained impedance spectra, for the electrodes “ACS” and “AC1.8S”, are shown in Figure 4 and the data were modeled by the EEC shown in Scheme 1.

Impedance spectra of the electrode “ACS” at pH 7 are shown in Figure 4a. Impedance spectra recorded in the presence of cupric ions revealed increasing of the charge-transfer resistance when compare to the value obtained at pH 5. This is in accordance to the formation of various copper hydroxide complexes on the electrode surface. The addition of cysteine has the same effect, as at pH 5.

Impedance spectra obtained for the electrode “AC1.8S” (Figure 4b) revealed an increase (Table 2) of the charge-transfer resistance R_1_, when compared to the values obtained at pH 5 (see Table 1). This is in accordance to the formation of the various copper hydroxo complexes at the electrode surface, which can reduce electron transfer reaction. By addition of cysteine, similar behavior and trends were observed as at pH 5.

### 3.3. Infrared Spectra of the Electrodes

**Figure 5:**
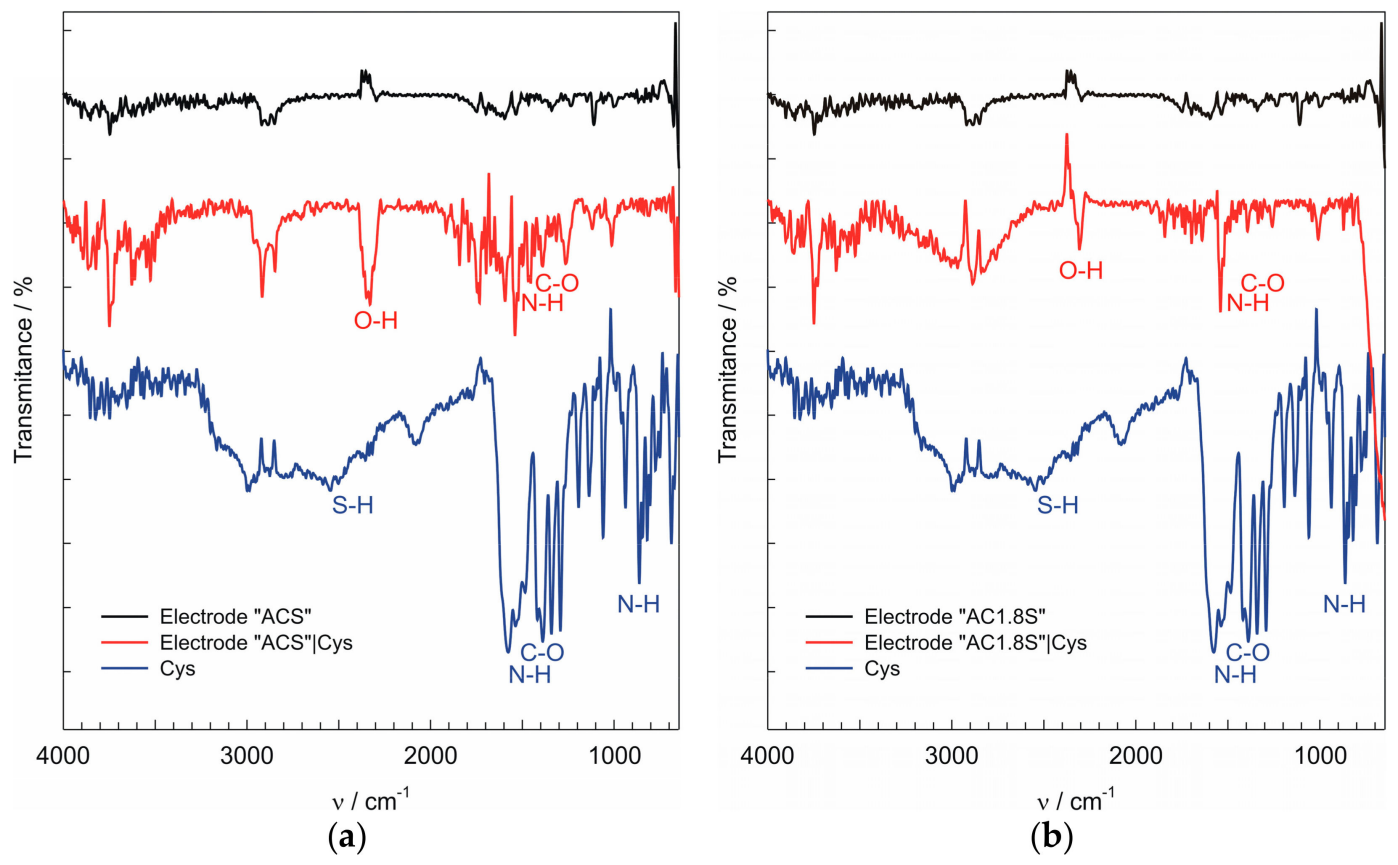
The IR spectra of the: (**a**) electrode “ACS”; (**b**) electrode “AC1.8S”. The surface of the electrodes (black line); Surface of the electrode after CVs was performed (red line); Cysteine powder (blue line).

To obtain additional information about processes that take place at the surface of the electrodes “ACS” and “AC1.8S”, IR spectra were recorded. As fouling of the electrode surfaces was more pronounced at pH 7, IR spectra have been recorded for the electrodes “ACS” and “AC1.8S” after the recording of cyclic voltammograms (at pH 7) in the presence of 1 × 10−4 M cysteine. As presented, the IR spectra confirm the binding of cuprous cysteinate on the surface of the electrodes “ACS” and “AC1.8S”. In Figure 5, the intense peaks at wavenumbers around 1564 cm−1 and 870 cm−1 were assigned to stretching vibration of the N–H bond. The peak at the wavenumber around 1423 cm−1 was assigned to symmetric COO− stretching [51]. In the IR spectra of the cysteine powder the peak at the wavenumber around 2540 cm−1 was assigned to the S-H bond. Lack of this peak in the IR spectra of the electrode surface indicates that the S-H bond was cleaved and that a new Metal-S bond was formed between the cysteine and the electrode “AC1.8S” [52].

### 3.4. Influence of Scan Rate on CVs of Electrode “ACS”

**Figure 6:**
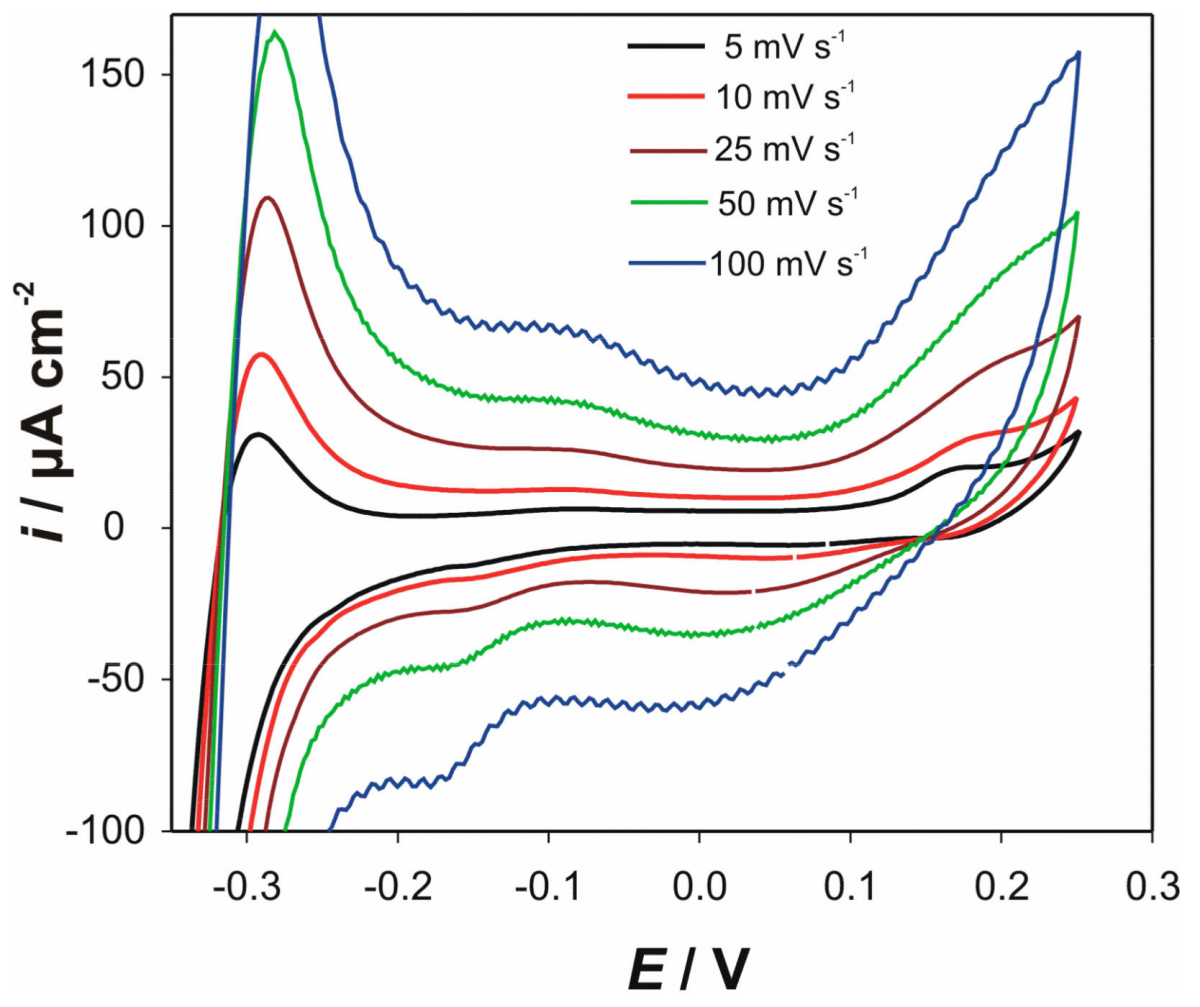
CVs of the electrode “ACS” in the presence of 1 × 10^−4^ M cysteine at various scan rates. Measurements were performed in 90 mL 0.1 KNO_3_ + 10 mL 0.1 M acetate buffer pH 5.

For understanding the undergoing electrochemical reactions, cyclic voltammograms at different scan rates (in the presence of the 1 × 10−4 M cysteine) were recorded at the electrodes “ACS” and “AC1.8S”, at both pH values. Similar psuedocapacitive behavior was obtained for both electrodes. As an example of obtained voltammograms, influence of the scan rate on CVs of the electrode “ACS” at pH 5, is presented in Figure 6.

### 3.5. Amperometric Response to Cysteine on the Electrode “ACS”

When compared to the other prepared electrodes, the electrode “ACS” showed significantly improved electroactivity (sensitivity) towards cysteine, at relatively lower positive operating potential. Also, pseudocapacitance behavior was less noticeable as well as in the case of the electrode “AC1.8S”. Thus, for the further investigation, as far as electrochemical determination of cysteine is concerned, the electrode “ACS” was chosen.

**Figure 7:**
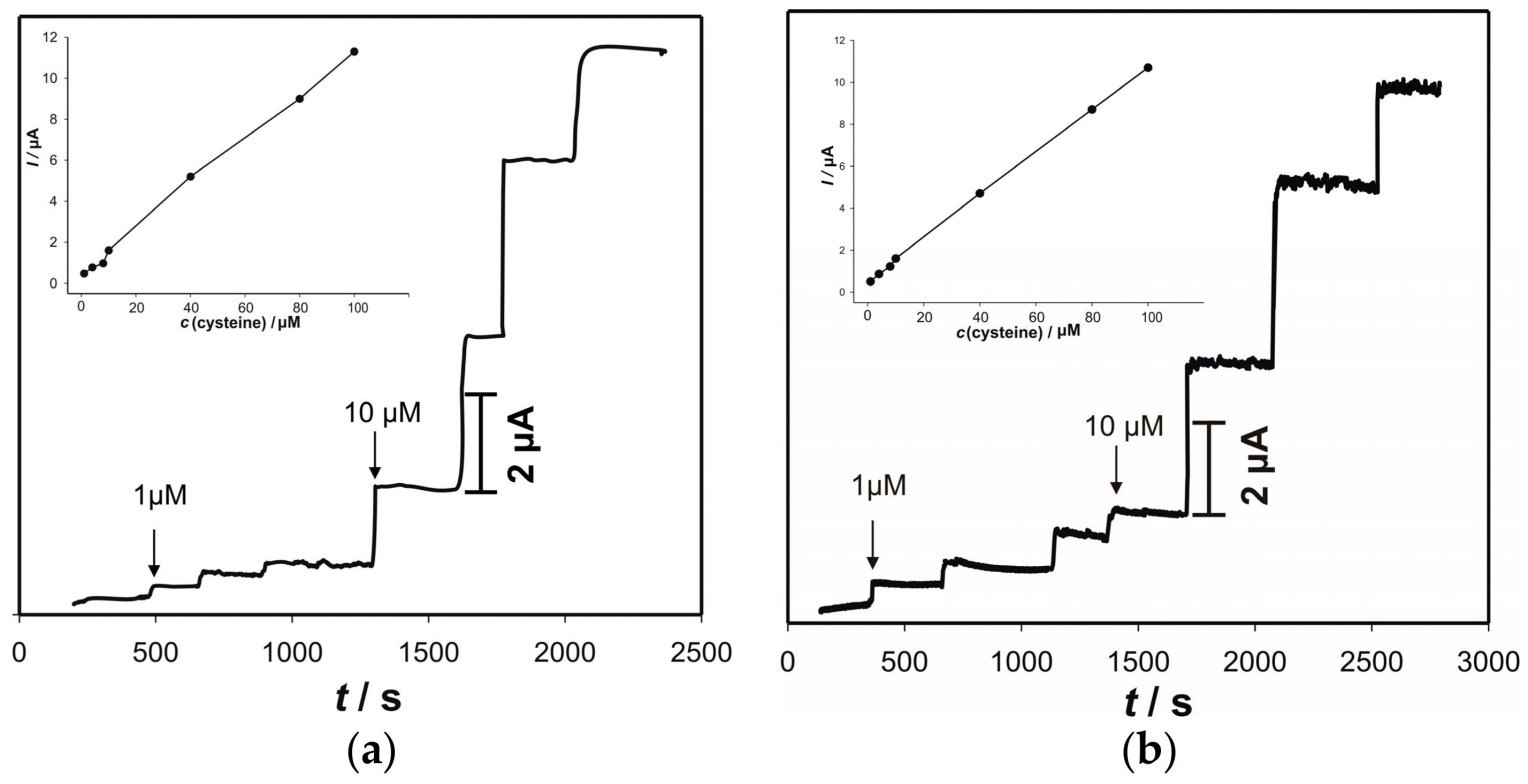
Amperometric responses of the electrode “ACS” on successive addition of cysteine. Measurements were performed in: (**a**) in 90 mL 0.1 KNO_3_ + 10 mL 0.1 M acetate buffer pH 5 at the 0.142 V; (**b**) in 90 mL 0.1 KNO_3_ + 10 mL 0.1 M phosphate buffer pH 7 at the 0.04 V. Insets show corresponding calibration plots.

Based on the preliminary results (CVs and EIS), as the best appropriate method for the electrochemical detection of cysteine, amperometry was chosen. Amperometric measurements were performed with constant stirring of the solution at two pH values. Two anodic potentials were chosen from the anodic potential window: 0.142 V for pH 5 and 0.04 V for pH 7. Figure 7 shows the results of the chronoamperometric measurements, obtained with the electrode “ACS” at the chosen anodic potential for successive addition of cysteine. These potentials were chosen to obtain higher reaction rate and stronger driving force, to minimize the fouling effect. The electrode “ACS” exhibits a quick response to the addition of cysteine at both pH values.

Some drifting in the current signal at pH 7, which is probably related to the surface fouling effect, can be noticed. The amperometric responses increase linearly with the increase of the cysteine concentrations that range from 1 μM to 100 μM (R = 0.995; *n* = 38). The sensitivities, at pH 5 and pH 7, were calculated to be 0.11 μA μM^−1^ and 0.10 μA μM^−1^, respectively. The limits of detection were (3σ: S/N = 3) determined to be 0.036 μM and 0.024 μM at both pH 5 and 7, respectively. Bearing in mind the physiological conditions as well as the lower oxidation potential, pH 7 was chosen for further studies.

**Figure 8:**
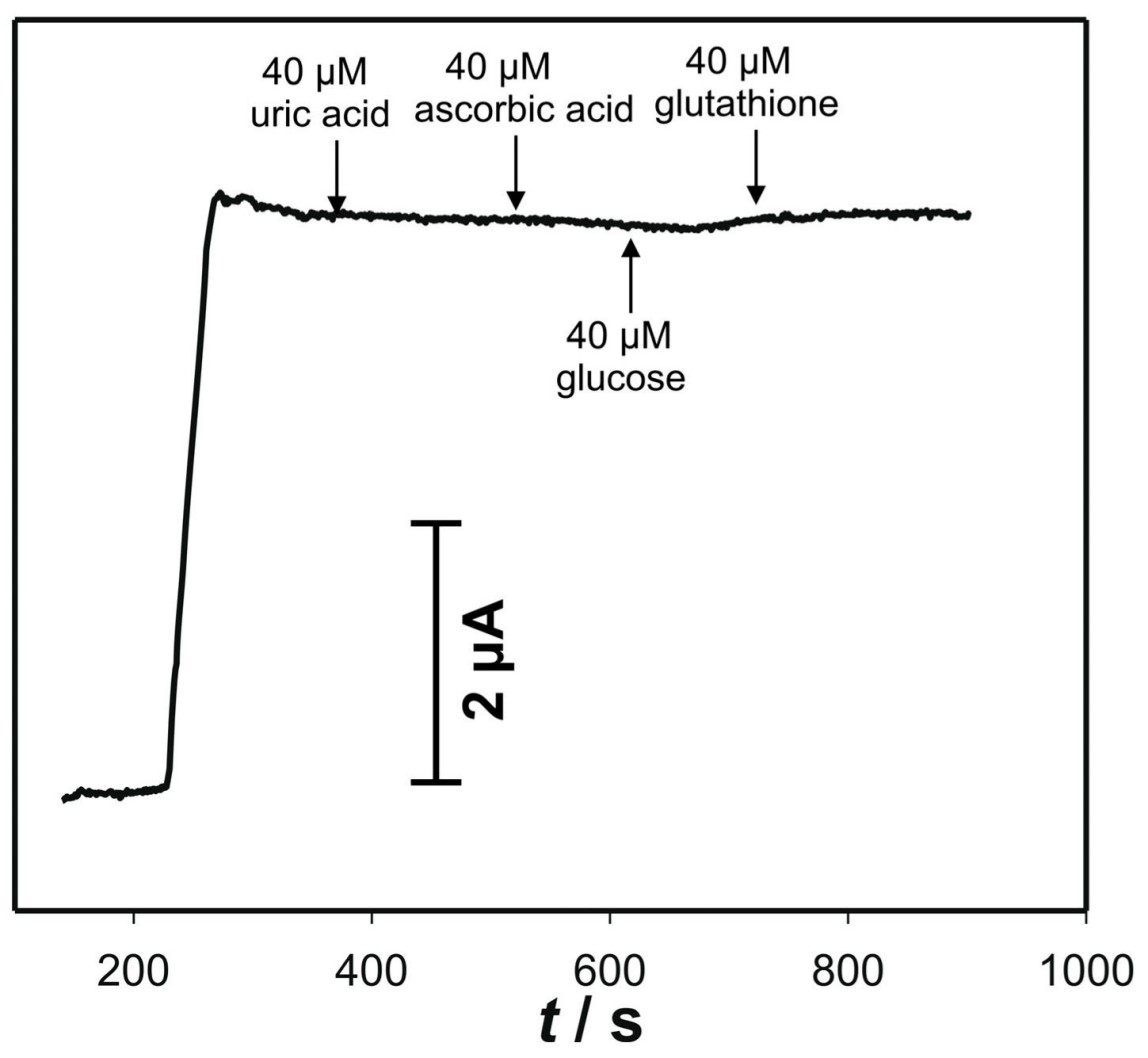
Amperometric responses of the electrode “ACS” to successive addition of the possible interferences. Initial concentration of cysteine was 30 μM. Measurements were performed in 90 mL 0.1 KNO_3_ + 10 mL 0.1 M phosphate buffer pH 7, at the 0.04 V.

Influence of various species on the determination of cysteine at pH 7, are presented in Figure 8. Obvious current response to the cysteine concentration of 30 μM can be noticed. On contrary, by the addition of uric acid, ascorbic acid, glucose, or even surprisingly glutathione, no noticeable current response was observed. This is mainly a consequence of the relatively low operating potential at pH 7.

The reproducibility of the electrode was investigated under pH 7 and in the presence of 30 μM cysteine. The relative standard deviation (RSD) of the five-value data set was 6%. The excellent long-term stability can be achieved, when the electrode is kept and treated properly. This means that it must be stored in a dark place and it must be mechanically treated (wiped or slightly polished with alumina 0.05) after seven measurements. Comparison of our electrode to other reported amperometric sensors for determination of cysteine, reported in literature, is shown in Table 3.

### 3.6. Application of the Cysteine Determination of in a Dietary Supplement

The practical usage of the designed electrode, in terms of percentage recovery, was investigated by the determination of a cysteine derivative (*N*-acetyl cysteine (NAC)) in NAC-Twinlab^®^ dietary supplement. Determination was performed at pH 7 using standard addition method. Results of the calculated recovery values are presented in Table 4. As shown, the recoveries were satisfactory, and the electrode has a potential to be used for the detection of cysteine (or its derivate) in real samples.

## 4. Conclusions

Robust, simple, and practical electrodes were constructed from different mixed silver-copper sulfides. Electrochemical behavior of the prepared electrodes revealed that the electrode “ACS” has the best electrocatalytical properties towards cysteine. These properties are characterized by the relatively low oxidation potentials at pH 5 and pH 7. Because of the oxidation, the electrode surface fouling effect was ascertained at the electrode “AC1.8S” and to a small extent at the electrode “ACS”. It is attributed to the formation of cystine together with the copper cysteinate complex. At the electrode “AC0.5S” this effect was not observed. For the electroanalytical determination of cysteine, the amperometric determination with the electrode “ACS” was chosen. Bearing in mind the physiological conditions and the low oxidation potential, the best amperometric response was obtained at pH 7 and the potential of +0.04 V. The electrode demonstrates excellent selectivity in the presence of various electroactive biomolecules. The designed electrode was successfully applied for the determination of cysteine in a dietary supplement with the satisfactory recovery. The fast response, together with the linear range and simplicity, makes this robust electrode a promising cysteine sensor.

## Figures and Tables

**Table 1:** Impedance parameters for electrodes “ACS” and “AC1.8S” (spectra recorded at pH 5), determined using the equivalent circuit, are presented in Scheme 1.

electrode “ACS”		**10^5^ × *Q*_1_/Ω^−1^ cm^−2^ s*^n^***	***n*_1_**	***R*_1_/Ω cm^2^**	**10^5^ × *Q*_2_/Ω^−1^ cm^−2^ s*^n^***	***n*_2_**
	no cysteine	12.72	0.853	713	25.02	0.629
	no cysteine but with 1 × 10^−5^ M Cu^2+^	11.12	0.843	66	53.85	0.293
	*c* (cys) = 1 × 10^−5^ M	7.99	0.879	126	46.25	0.379
	*c* (cys) = 5 × 10^−5^ M	9.87	0.849	648	29.18	0.590
	*c* (cys) = 1 × 10^−4^ M	12.68	0.841	979	21.66	0.613
electrode “AC1.8S”		**10^7^ × *Q*_1_/Ω^−1^ cm^−2^ s*^n^***	***n*_1_**	***R*_1_/kΩ cm^2^**	**10^5^ × *Q*_2_/Ω^−1^ cm^−2^ s*^n^***	***n*_2_**
	no cysteine	0.20	0.896	5.3	10.42	0.651
	no cysteine but with 1 × 10^−5^ M Cu^2+^	0.10	0.953	5.3	18.06	0.202
	*c* (cys) = 1 × 10^−^^4^ M	0.11	0.950	7.9	17.92	0.182
	*c* (cys) = 5 × 10^−^^5^ M	0.14	0.928	10.2	15.40	0.460
	*c* (cys) = 1 × 10^−^^4^ M	0.13	0.930	11.7	10.40	0.650

**Table 2:** Impedance parameters for electrodes “ACS” and “AC1.8S” (spectra recorded at pH 7), determined using the equivalent circuit, are presented in Scheme 1.

electrode “ACS”		**10^5^ × *Q*_1_/Ω^−1^ cm^−2^ s*^n^***	***n*_1_**	***R*_1_/Ω cm^2^**	**10^5^ × *Q*_2_/Ω^−1^ cm^−2^ s*^n^***	***n*_2_**
	no cysteine	15.85	0.824	619	52.43	0.580
	no cysteine but with 1 × 10**^−^**^5^ M Cu^2+^	12.94	0.846	317	104.72	0.450
	*c* (cys) = 1 × 10**^−^**^5^ M	11.36	0.857	353	97.87	0.484
	*c* (cys) = 5 × 10**^−^**^5^ M	10.84	0.857	515	62.95	0.623
	*c* (cys) = 1 × 10**^−^**^4^ M	15.85	0.824	619	52.43	0.580
electrode “AC1.8S”		**10^7^ × *Q*_1_/Ω^−1^ cm** ^−**2**^ **s*^n^***	***n*_1_**	***R*_1_/kΩ cm^2^**	**10^5^ × *Q*_2_/Ω** ^−**1**^ **cm** ^−**2**^ **s*^n^***	***n*_2_**
	no cysteine	0.12	0.945	16.0	16.69	0.666
	no cysteine but with 1 × 10^−5^ M Cu^2+^	0.12	0.942	11.3	25.22	0.366
	*c* (cys) = 1 × 10^−4^ M	0.12	0.942	12.9	22.26	0.378
	*c* (cys) = 5 × 10^−5^ M	0.12	0.940	14.6	14.30	0.605
	*c* (cys) = 1 × 10^−4^ M	0.11	0.948	15.4	14.08	0.496

**Table 3:** Comparison of the analytical performances of various reported electrodes for the amperometric determination of cysteine.

Electrode	*E* (V) vs. Ag/AgCl	LOD (μM^−1^)	Linear Range (μM^−1^)	Sensitivity	Ref.
Carbon paste electrode modified with 4-nitrophthalonitrile	0.33	0.25	0.8–13.2	37 nA μM^−1^	[3]
Bulk carbon electrodes modified with cobalt phthalocyanine	0.40	0.2	1–12	8.89 nA μM^−1^	[53]
Glassy carbon modified with Nile blue	−0.45	1.3	10–250	not reported	[54]
Glassy carbon electrode modified with MWCNTs	0.18	5.4	10–500	3 nA μM^−1^	[55]
Oxidation product of guanine at ZnOx nanoparticles modified GCE	0.50	0.05	0.3–20	28.5 nA μM^−1^	[22]
Boron-doped carbon nanotube modified GCE	0.47 vs. SCE	0.26	0.78–200	0.025 nA μM^−1^	[56]
Pt nanoparticles/poly(o-aminophenol) film on GCE	0.41	0.08	0.4–6300	not reported	[21]
Positively charged poly(diallyldimethylammonium chloride) and negatively charged MWNTs on glassy carbon	0.80	0.3	20–1300	not reported	[57]
Au/CeO_2_ composite nanofibers on screen printed electrodes	0.7	0.01	to 200	321 μA mM^–1^ cm^–2^	[58]
Cerium oxide nanofibers modified screen printed carbon electrode	0.7	0.02	to 200	120 μA mM^–1^ cm^–2^	[59]
Palladium nanoparticles grown on reduced graphene oxide in a Nafion matrix	0.6 vs. SCE		0.5–10	1.3 μA mM^–1^ cm^–2^	[60]
AuNPs/SG-PEDOT/GCE	0.32	0.02	0.1–382	25.85 μA mM^–1^ cm^–2^	[61]
NiO NPs/N-rGO/CPE	0.65	0.1	0.3–1620	0.051 μA mM^–1^	[62]
mixed silver-copper sulfides	0.04	0.024	1–100	0.1 μA mM^–1^	our work

**Table 4:** Determination of cysteine in a dietary supplement with the electrode “ACS”, at pH 7 (90 mL 0.1 KNO_3_ + 10 mL 0.1 M phosphate buffer), by using the standard addition method. Recovery was calculated based on the five measurements.

Sample No.	Original Content/μM	Added/μM	Found/μM	Recovery/(%)
1	1	-	0.92	
2	10	10	20.15	100.8
3	10	20	30.09	100.3
4	10	30	39.56	98.9
5	10	40	49.85	99.7
